# A novel mutation in *FRMD7* causing X-linked idiopathic congenital nystagmus in a large family

**Published:** 2008-01-11

**Authors:** Xiang He, Feng Gu, Yujing Wang, Jinting Yan, Meng Zhang, Shangzhi Huang, Xu Ma

**Affiliations:** 1 Department of Genetics, National Research Institute for Family Planning, Beijing, China; 2 Peking Union Medical College, Beijing, China; 3 Dezhou Woman and Child Hospital, Beijing, China; 4 Department of Medical Genetics, Institute of Basic Medical Sciences, Chinese Academy of Medical Sciences, Beijing, China; 5 Department of Reproductive Genetics, WHO Collaborative Center for Research in Human Reproduction, Beijing, China

## Abstract

**Purpose:**

To identify the gene responsible for causing an X-linked idiopathic congenital nystagmus (XLICN) in a six-generation Chinese family.

**Methods:**

Forty-nine members of an XLICN family were recruited and examined after obtaining informed consent. Affected male individuals were genotyped with microsatellite markers around the *FRMD7* locus. Mutations were comprehensively screened by direct sequencing using gene specific primers. An X-inactivation pattern was investigated by X chromosome methylation analysis.

**Results:**

The patients showed phenotypes consistent with XLICN. Genotype analysis showed that male affected individuals in the family shared a common haplotype with the selected markers. Sequencing *FRMD7* revealed a G>T transversion (c.812G>T) in exon 9, which caused a conservative substitution of Cys to Phe at codon 271 (p.C271F). This mutation co-segregated with all affected individuals and was present in the obligate, non-penetrant female carriers. However, the mutation was not observed in unaffected familial males or 400 control males. Females with the mutant gene could be affected or carrier and they shared the same inactivated X chromosome harboring the mutation in blood cells, which showed there is no clear causal link between X-inactivation pattern and phenotype.

**Conclusions:**

We identified a novel mutation in *FRMD7* and confirmed the role of this mutation in the pathogenesis of X-linked congenital nystagmus.

## Introduction

Congenital nystagmus (CN) is a common oculomotor disorder (frequency of 1/1,500 live births) characterized by bilateral involuntary, periodic, predominantly ocular oscillations. CN onset typically occurs at birth or within the first few months of life [1] and occurs secondary to the genetic ocular diseases such as albinism, achromatopsia, and Leber congenital amaurosis (OMIN 204000).

CN can be an idiopathic disease or associated with various diseases as a syndrome [2]. The inheritance model is mainly X-linked idiopathic congenital nystagmus (XLICN), but autosomal recessive (OMIN 257400) and autosomal dominant (OMIN 164100,608345,193003) forms have been described.

Some studies indicated that two disease loci of XLICN were mapped to Xq26-q27 and Xp11.4- Xp11.3 [1,3]. Recently, Tarpey et al. [4-6] identified several mutations in *FRMD7* (OMIN 300628), a gene localizing to Xq26-q27 and responsible for a major part of XLICN.

**Figure 1:**
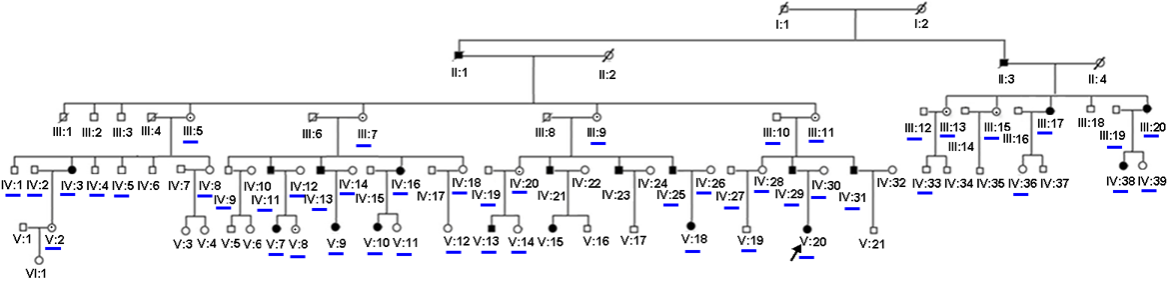
Analysis of methylation of *Hpa*II sites in the human androgen-receptor locus. The different alleles at the X androgen-receptor locus are shown. The upper is the results of DNA without treatment of *Hpa*II. The bottom shows that only genomic DNA of the female affected individuals (V:20) and two carriers (III:9 and III:11) were digested by *Hpa*II. As to affected female (V:20), after digestion, only an amplified band of AR allele from her mother has been achieved and she may inherit the mutant allele of *FRMD7* on the active X chromosome (unmethylated) from the father. While, carrier (III:11) and her sister (III:9, carrier) hold a different active X chromosome. Affected male individual (IV:29) and his affected brother (IV:31) inherited different allele on the AR locus from their mother (III:11).

In this study, 49 members in a Chinese XLICN family were recruited and examined. Male affected members were genotyped with microsatellite markers at *FRMD7*, and direct sequencing identified a mutation. Using one female patient, two carriers, and other family members in this large family (Figure 1), we assessed the correlation between X-inactivation pattern and phenotype.

## Methods

### Clinical evaluations and DNA specimens

**Figure 2:**
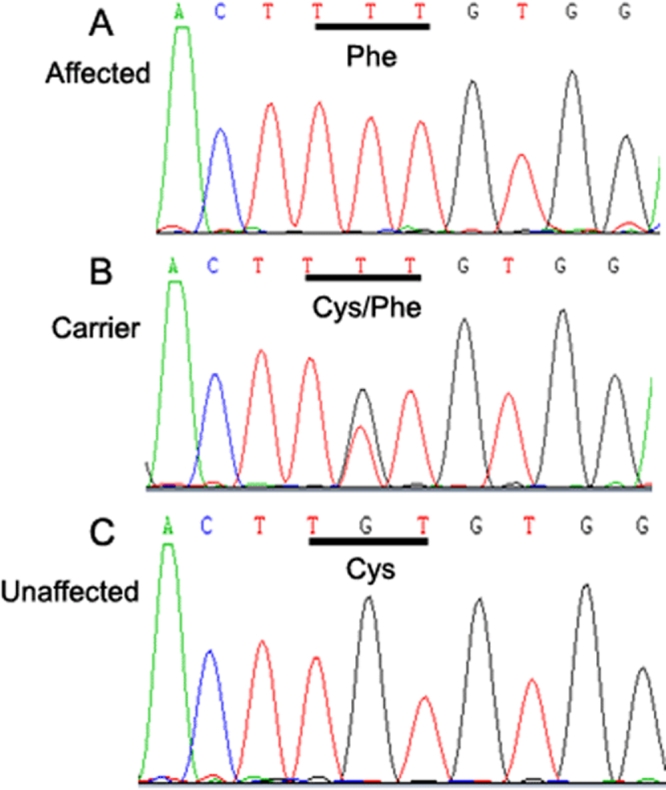
The Chinese X-linked idiopathic congenital nystagmus pedigree. The squares and circles symbolize males and females, respectively. Dots in the middle of the circle denote that the female is a carrier. Black and white denotes affected and unaffected status, respectively. An individual is identified (ID) by generation number and the aforementioned symbols. ID underscored with blue indicates individuals enrolled in this study.

This study followed the tenets of the Declaration of Helsinki, and the protocol was approved by the Ethics Committee at the National Research Institute for Family Planning. Informed consent was obtained from all family members participating in this study. The family, originating from Shandong province in China, contained 21 affected individuals within a six-generation pedigree (Figure 2). In total, 49 members in this family were recruited, including 15 affected individuals (5 males and 10 females) and 34 unaffected individuals or spouses (Figure 2). Ophthalmologists confirmed the diagnosis of CN and there was no history of other ocular or systemic abnormalities in the family. A 5 ml venous blood sample was drawn into an ethylenediamine tetraacetic acid (EDTA) sample tube from every subject.

### Genotyping and allele-sharing analysis

Genotyping was performed as described previously [7]. The primer sequences were taken from GDB. Allele-sharing analysis was performed with two microsatellite markers, DXS1047 and DXS691, which were linked with *FRMD7* [4] on five affected males individuals. The physical locations of DXS1047-*FRMD7*-DXS691 are 128.9 Mb, 131.0 Mb, and 135.2 Mb, respectively.

### DNA sequencing

Mutations in *FRMD7* (NM_194277) were screened by direct sequencing. Polymerase chain reaction (PCR) products of the 12 exons and flanking intron sequences of *FRMD7* were sequenced on an ABI A3730 Automated Sequencer (Applied Biosystem, Foster City, CA) [5].

### Denaturing HPLC

Wave DHPLC (Transgenomic, San Jose, CA) was used to screen exon 9 of *FRMD7* from patients, carriers, family members, and 400 normal, unrelated, male individuals. DHPLC was performed according to the protocols described previously [7] with initial concentrations of buffer A (0.1 M triethylammonium acetate-TEAA) at 53% and 47% for buffer B (0.1 M TEAA containing 25% acetonitrile) while maintaining the procedure at 58.7 °C.

### X-Inactivation assay

Genomic DNA (1 μg) from a normal male (III:10, as negative control), two male patients (IV:29 and IV:30, as the positive controls), a female patient (V:20), and two obligate non-penetrant carriers (III:9 and III:11) was incubated overnight with and without 1 U *Hpa*II in a total volume of 20 μl. After a 1:2 dilution with water, 2 μl of the diluted digest was amplified by PCR to detect the polymorphic CAG repeat of the androgen receptor (*AR*). PCR products were separated on a 6% polyacrylamide denaturing gel for 1.5 h using constant voltage (600 V) and were subsequently detected by silver stain.

## Results

### Clinical data

In this family, there were nine normal carriers and 11 affected females (Figure 2), who have a milder phenotype than the affected males. All affected individuals had nystagmus in early childhood. Night blindness or photophobia were not observed in any of the affected individuals in the family nor were there any incidences of systemic or other ocular anomalies. The X-linked disease was transmitted from female carriers/patients to afflicted sons yet there was no evidence of male-to-male transmission in this family.

The proband was a 10-year-old female (V:20, Figure 2). Her visual acuity was about 20/25. While visual acuity of her father (36-year-old, IV:29, also affected) is 20/32 (right/left). Myopia was also identified in her father (right/left, 27.50 mm/27.20 mm).

### Allele-sharing analysis

Allele-sharing analysis confirmed the linkage of the disease in the family with the mutation in *FRMD7* (data not shown).

### Mutation detection in *FRMD7*

**Figure 3:**
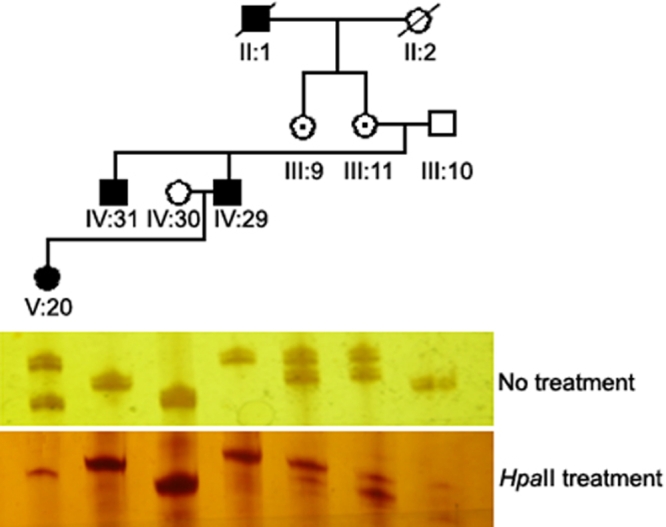
DNA sequence chromatograms. DNA sequence chromatograms of the affected members (**A**), carriers (**B**), and unaffected members (**C**) in an X-linked idiopathic congenital nystagmus family is shown. There is a single base G>T transversion in exon 9 of *FRMD7* that causes a conservative substitution of Cys to Phe at codon 271 (p.C271F). This mutation co-segregated with all affected individuals and was present in the obligate, non-penetrant carrier.

Sequence analysis revealed a G>T transversion in exon 9 (Figure 3) that caused a conservative substitution of Cys to Phe at codon 271 (p.C271F). No sequence change was detected in the remaining coding sequence of *FRMD7*. DHPLC analysis confirmed this mutation and found the mutation to co-segregate with all affected individuals and obligate carriers in the family. No mutation was detected in any of the unaffected male family members or in any of the 400 normal unrelated male individuals.

### Multiple-sequence alignment and mutation analysis

Using the National Center for Biotechnology Information (NIH) websites, a multiple-sequence alignment of the FRMD7 proteins in various species (*Homo sapiens*, *Canis familiaris*, *Mus musculus, Rattus norvegicus*, and *Gallus gallus*) was obtained. This alignment was compared to homologous proteins (FERM, RhoGEF, FARP2 protein) using DNAMAN biosoftware (Lynnon Biosoft, Quebec, Canada). The mutation, p.C271F, occurred within a highly conserved region of the gene. The online bioinformatics software SIFT (Sorting Intolerant, Tolerant) algorithm [8] predicted whether the amino acid substitution in FRMD7 would have a phenotypic effect. The software determined that the substitution of Cys to Phe at position 271 was deleterious.

The predicted p.C271F substitution represented a conservative amino-acid change with the uncharged polar sulfhydryl side group of Cys replaced by the nonpolar group on Phe. This change is likely to destabilize the protein by inducing structural changes by placing a larger amino acid within a restricted space of the protein. Additional changes associated with losing the free sulfhydryl group may disturb the disulfide bond formation between inter- or intra-molecular residues.

Overall, the cosegregation of the G-T transversion is only found within the affected and obligate, non-penetrant carriers in the pedigree. These results suggest that the p.C271F substitution was a causative mutation.

### X-Inactivation assay

Affected female individuals carried the mutated X chromosome. Genes in this affected X chromosome were active and unmethylated because the DNA could be digested with *Hpa*II (V:20, the allele from her father IV:29 is cut by *Hpa*II). As a result, only methylated DNA on the X chromosome was used as a PCR template (Figure 1, V:20 and the allele from her mother, IV:30). The X chromosome from carrier III:9 was methylated and contained the mutated *FRMD7*. Additionally, carrier III:11 had an unmethylated X chromosome containing the *FRMD7* mutation. Take together, carrier (III:11) and her sister (III:9, carrier) held a different active X chromosome. Meanwhile, affected male individual (IV:29) and his affected brother (IV:31) inherited different allele on the AR locus from their mother (III:11).

## Discussion

Congenital nystagmus is a clinically and genetically heterogeneous disease that causes visual impairment in childhood. Clinically, the congenital nystagmus is divided into idiopathic and nystagmus-related syndromes. Idiopathic congenital nystagmus is thought to represent an abnormal development of the ocular motor areas of the brain that control fixation. As these patients may have normal visual acuity, it is presumed that the nystagmus represents a primary defect in the parts of the brain responsible for ocular motor control.

Genetically, at least four loci have been proposed for familial idiopathic congenital nystagmus [1-6,9-12]. Two loci have been identified for XLICN with one being mapped to Xq26-q27 by Kerrison and the other to Xp11.4-p11.3 by Cabot [1,3].

In this family, it was obvious that the trait is X-linked because there was no male-to-male transmission, but there was frequent female-to-male transmission. The estimated penetrance among obligate female carriers was estimated to be 55% [11 of 20] with no age dependent affect. The penetrance in this family is lower than that found in a family studied by Schorderet and colleagues [5]. The different penetrance in different XLICN families is consistant with previous studies by Waardenburg. He felt there was no reason to separate an X-linked recessive from an X-linked dominant form as some have attempted (OMIM 310700). In some families, the disorder is recessive in one line and dominant in another. The explanation could be that the mutation is identical, but a series of “wildtype” isoalleles have different effects on penetrance of the mutation in the heterozygous female.

Meanwhile, X chromosome inactivation may be one mechanism for the variation in penetrance. Inactivation is a methylation-dependent phenomenon and consists of transcriptional silencing of one of the two alleles on X chromosomes in mammalian females [13]. Most females are mosaics with a mixture of cells expressing either their mothers or fathers X-linked genes. Often, cell mosaicism is advantageous by ameliorating the deleterious effects of X-linked mutations and contributing to physiologic diversity [14]. Yet, in some cases, females carrying the mutant gene have clinical manifestation as observed in this study.

To uncover the correlation between the X-inactivation pattern and phenotype, we assayed for the pattern in one female patient, two carriers, and other family members in this large family. Because the affected IV:29 and IV:31 patients inherited different alleles of CAG repeats at *AR* from their mother (III:11), the results showed that there is at least one recombination event in these nuclear families. Since physical distance from the *FRMD7* to the *AR* gene is 64.2 Mb (131.0 Mb, 66.8 Mb for *FRMD7*, *AR*, respectively), a marker closer to the *FRMD7* locus is required to assay the methylation status of the wild-type and mutant allele.

Carriers III:9 and III:11 showed different methylation patterns for the X chromosome, implying that a molecular basis for variable methylation might not be involved in the dissimilar penetrance in this family. Female heterozygotes contain two cell types where the proportion of cells departs from equality following cell selection at the tissue or organism level [15]. Willemsen et al. [16] reported that monozygotic twin sisters with the fragile X mutation could have different phenotypes resulting from skewed X-inactivation. Furthermore, other results have proven that familial non-random inactivation is linked to the X-inactivation center in heterozygotes that manifest hemophilia A, one of the inherited blood diseases [17]. While the patterns of X-inactivation in blood may not necessarily reflect those in tissue, the methylation status of *FRMD7* in the pathologic tissue could be different from that of blood. Taken together, no correlation between the X-inactivation pattern and phenotype were observed in blood analyzed in this study. This finding is consistent with a recent study [18] and as such, further studies need to investigate the X-inactivation pattern and phenotype in this family.

Since only two loci have been mapped on the X chromosome and the results of genotyping show that the affected individuals shared the same allele with the *FRMD7* locus, we sequenced the coding and flanking intron sequences of *FRMD7*, the only reported gene responsible for XLICN. This data led us to conclude that the family in this study contains a novel mutation.

Tarpey et al. [4] identified 22 novel mutations in *FRMD7* in 26 families with XLICN. Schorderet et al. [5] identified five novel mutations in six families with XLICN. Among these 22 mutations, a missense mutation of C271Y in exon 9 was detected in a Scottish family. In our study, the p.C271F mutation is responsible for this disease. Schorderet et al. [5] identified another mutation (H275P) in exon 9. These studies suggested that the coding region of exon 9 is critical to the normal function of the FRMD7 protein.

*FRMD7* encodes a member of the FERM-domain (Band 4.1 family) containing proteins. The FERM domain is found in cytoskeletal-associated proteins such as ezrin, moesin, radixin, 4.1R, and merlin. Proteins of the FERM-domain family are thought to be involved in cytoskeleton attachment to the plasma membrane [19,20]. The mouse genome encodes at least 50 FERM proteins, but the functions of only a few, including the actin-binding proteins ezrin (also known as Vil2), radixin, moesin, Nf2 (also known as merlin), and the erythrocyte protein band 4.1, have been characterized. Therefore, from the function of these FERM proteins, FRMD7 may have functions relevant to cell structure, cell migration, normal cell growth, cellular differentiation, and signal transduction.

*FRMD7* is expressed in early human embryos at about 56 days post-ovulation in the ventricular layer of the forebrain, midbrain, cerebellar primordium, spinal cord, and the developing neural retina [4]. This restricted expression suggests a specific role in the control of eye movement and gaze stability.

This study confirmed that *FRMD7* plays an important role in motor control of eye movement and provided additional information on gene mutations in exon 9 of *FRMD7* that lead to mutations causing XLICN. Therefore, *FRMD7* is an ideal candidate for XLICN mutation screening. When no mutation has been identified, it is suggested to scan the whole X chromosome to mapping the responsible gene. While the role of *FRMD7* in disease genesis is still unclear, further studies need to provide insights into the molecular pathology of XLICN.
